# [2,6-Bis(diphenyl­phosphino­oxy)phen­yl]bis­(trimethyl­phosphine)cobalt(I)

**DOI:** 10.1107/S160053681001634X

**Published:** 2010-05-12

**Authors:** Zhe Lian, Guoqiang Xu, Xiaoyan Li

**Affiliations:** a School of Chemistry and Chemical Engineering, Shandong University, Shanda Nanlu 27, Jinan 250100, People’s Republic of China

## Abstract

The title compound, [Co(C_30_H_23_O_2_P_2_)(C_3_H_9_P)_2_], was synthesized by the addition of a Co(PMe_3_)_4_ solution to (PPh_2_O)_2_C_6_H_4_. The Co^I^ atom displays a trigonal-bipyramidal geometry with the two P atoms of the ‘PCP’ pincer ligand and the P atom of one of the trimethyl phosphine ligands forming the basal plane, whereas the metalated C atom and the P atom of the second phospine ligand occupy the apical sites. The Co—C distance is 1.961 (2) Å and the C—Co—P angle is 171.96 (6)°.

## Related literature

For uses of ‘PCP’ pincer complexes, see: Boom & Milstein (2003 ▶); Bedford *et al.* (2006 ▶); Gomez-Benitez *et al.* (2006 ▶); Aydin *et al.* (2007 ▶); Kimura & Uozumi (2006 ▶); Xu *et al.* (2009 ▶).
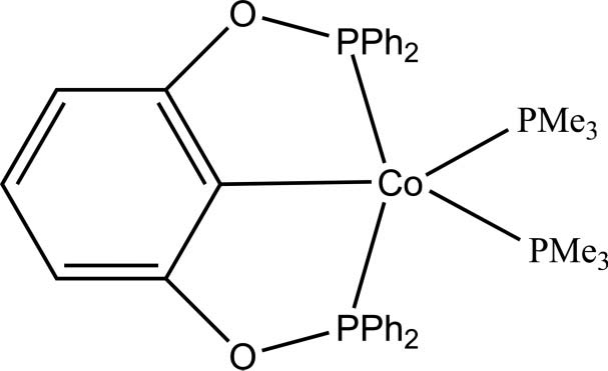


## Experimental

### 

#### Crystal data

[Co(C_30_H_23_O_2_P_2_)(C_3_H_9_P)_2_]
*M* *_r_* = 688.50
Monoclinic, 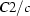

*a* = 31.437 (6) Å
*b* = 13.344 (3) Å
*c* = 19.187 (4) Å
β = 123.85 (3)°
*V* = 6685 (3) Å^3^
*Z* = 8
Mo *K*α radiation
μ = 0.74 mm^−1^
*T* = 293 K
0.20 × 0.15 × 0.10 mm

#### Data collection

Bruker SMART CCD area-detector diffractometer
Absorption correction: multi-scan (*SADABS*; Sheldrick, 2004 ▶) *T*_min_ = 0.867, *T*_max_ = 0.930
25414 measured reflections
7056 independent reflections
6078 reflections with *I* > 2σ(*I*)
*R*_int_ = 0.073

#### Refinement

*R*[*F* ^2^ > 2σ(*F* ^2^)] = 0.039
*wR*(*F* ^2^) = 0.106
*S* = 1.03
7056 reflections
394 parameters
H-atom parameters constrained
Δρ_max_ = 0.55 e Å^−3^
Δρ_min_ = −0.63 e Å^−3^

### 

Data collection: *SMART* (Bruker, 1997 ▶); cell refinement: *SAINT* (Bruker, 1997 ▶); data reduction: *SAINT*; program(s) used to solve structure: *SHELXS97* (Sheldrick, 2008 ▶); program(s) used to refine structure: *SHELXL97* (Sheldrick, 2008 ▶); molecular graphics: *ORTEPIII* (Burnett & Johnson, 1996 ▶) and *ORTEP-3 for Windows* (Farrugia, 1997 ▶); software used to prepare material for publication: *SHELXTL* (Sheldrick, 2008 ▶).

## Supplementary Material

Crystal structure: contains datablocks I, global. DOI: 10.1107/S160053681001634X/dn2559sup1.cif

Structure factors: contains datablocks I. DOI: 10.1107/S160053681001634X/dn2559Isup2.hkl

Additional supplementary materials:  crystallographic information; 3D view; checkCIF report

## Figures and Tables

**Fig. 1.**
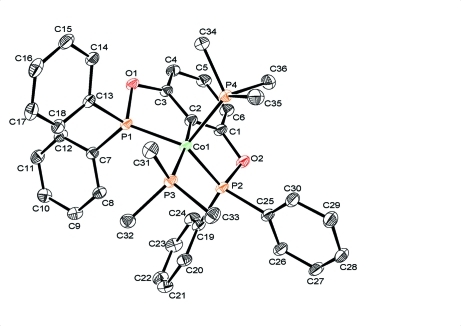
The asymmetric unit of (I) with the atom-labelling scheme. Displacement ellipsoids are drawn at the 30% probability level. H atoms have been omitted for clarity

## supplementary crystallographic information

### Comment

'PCP' pincer complex have attarcted much attention owing to their catalytic activities (Boom & Milstein, 2003; Bedford *et al.*, 2006; Gomez-Benitez *et al.*, 2006; Aydin *et al.*, 2007; Kimura & Uozumi, 2006). We previouly reported that the central sp3 C—H bond of (Ph_2_POCH_2_)_2_CH_2_ could be activated by Co(PMe_3_)_4_ to afford metallated 'PCP' pincer compounds at room temperature (Xu *et al.* 2009). Carrying on our investigations we explored the reaction of (PPh_2_O)_2_C_6_H_4_ with Co(PMe_3_)_4_, which afforded the title compound via C-H oxidative addition. Although the yield was only 30%, it was the only product which could be isolated and characterized. We proposed that a Co-H intermediate might be generated first, then the Co-H could be cleaved with the loss of hydrogen atom, affording the title compound with 18 e structure. However, the products resulting from the cleavage of the Co-H has not been isolated.

In the title compound, the cobalt atom displays a trigonal bipyramidal geometry with the two phosphorus of the PCP ligand and the phosphorus of one of the trimethyl phosphine ligand forming the basal plane whereas the metalated C atom and the phosphorus of the second phospine occupying the apex (Fig. 1). The Co1-C2 distance is 1.961 (2) Å and the C2-Co1-P3 angle is 171.96 (6)°.

### Experimental

Standard vacuum techniques were used in manipulations of volatile and air sensitive material. The title compound was synthesized by combining a solution of 1,3-Bis(diphenylphosphinooxy)benzene (920 mg, 2.00 mmol) in 40 ml of diethyl ether with a sample of Co(C_3_H_9_P) (720 mg, 2.00 mmol) in 30 ml of diethyl ether at 273 K. After kept stirring for 48 h at room temperature, the color changed from red to brown. Volatiles were concentrated and filtrated. Red crystals, which were suitable for X-ray diffraction, could be obtained from diethyl ether at 255 K.

### Refinement

All H atoms attached to C atoms were fixed geometrically and treated as riding with C—H = 0.96 Å (methyl) or 0.93 Å (aromatic) with U_iso_(H) = 1.2U_eq_(aromatic) or U_iso_(H) = 1.5U_eq_(methyl).

### Crystal data

**Table:**

[Co(C_30_H_23_O_2_P_2_)(C_3_H_9_P)_2_]	*F*(000) = 2880
*M**_r_* = 688.50	*D*_x_ = 1.368 Mg m^−^^3^
Monoclinic, *C*2/*c*	Melting point: 385 K
Hall symbol: -C 2yc	Mo *K*α radiation, λ = 0.71073 Å
*a* = 31.437 (6) Å	Cell parameters from 25414 reflections
*b* = 13.344 (3) Å	θ = 1.6–26.8°
*c* = 19.187 (4) Å	µ = 0.74 mm^−^^1^
β = 123.85 (3)°	*T* = 293 K
*V* = 6685 (3) Å^3^	Block, brown
*Z* = 8	0.20 × 0.15 × 0.10 mm

### Data collection

**Table:**

Bruker SMART CCD area-detector diffractometer	7056 independent reflections
Radiation source: fine-focus sealed tube	6078 reflections with *I* > 2σ(*I*)
graphite	*R*_int_ = 0.073
φ and ω scans	θ_max_ = 26.8°, θ_min_ = 1.6°
Absorption correction: multi-scan (*SADABS*; Sheldrick, 2004)	*h* = −39→39
*T*_min_ = 0.867, *T*_max_ = 0.930	*k* = −16→16
25414 measured reflections	*l* = −24→24

### Refinement

**Table:**

Refinement on *F*^2^	Primary atom site location: structure-invariant direct methods
Least-squares matrix: full	Secondary atom site location: difference Fourier map
*R*[*F*^2^ > 2σ(*F*^2^)] = 0.039	Hydrogen site location: inferred from neighbouring sites
*wR*(*F*^2^) = 0.106	H-atom parameters constrained
*S* = 1.03	*w* = 1/[σ^2^(*F*_o_^2^) + (0.0669*P*)^2^ + 0.8607*P*] where *P* = (*F*_o_^2^ + 2*F*_c_^2^)/3
7056 reflections	(Δ/σ)_max_ = 0.002
394 parameters	Δρ_max_ = 0.55 e Å^−^^3^
0 restraints	Δρ_min_ = −0.63 e Å^−^^3^

### Special details

**Table:**

Geometry. All esds (except the esd in the dihedral angle between two l.s. planes) are estimated using the full covariance matrix. The cell esds are taken into account individually in the estimation of esds in distances, angles and torsion angles; correlations between esds in cell parameters are only used when they are defined by crystal symmetry. An approximate (isotropic) treatment of cell esds is used for estimating esds involving l.s. planes.
Refinement. Refinement of *F*^2^ against ALL reflections. The weighted *R*-factor wR and goodness of fit *S* are based on *F*^2^, conventional *R*-factors *R* are based on *F*, with *F* set to zero for negative *F*^2^. The threshold expression of *F*^2^ > σ(*F*^2^) is used only for calculating *R*-factors(gt) etc. and is not relevant to the choice of reflections for refinement. *R*-factors based on *F*^2^ are statistically about twice as large as those based on *F*, and *R*- factors based on ALL data will be even larger.

### Fractional atomic coordinates and isotropic or equivalent isotropic displacement parameters (Å^2^)

**Table:**

	*x*	*y*	*z*	*U*_iso_*/*U*_eq_	
Co1	0.141627 (10)	0.22511 (2)	0.188845 (16)	0.02454 (9)	
P1	0.077347 (19)	0.18917 (4)	0.19360 (3)	0.02587 (12)	
P2	0.15209 (2)	0.33637 (4)	0.11980 (3)	0.02792 (12)	
P3	0.14305 (2)	0.08689 (4)	0.12942 (3)	0.02800 (12)	
P4	0.21421 (2)	0.20712 (4)	0.31383 (3)	0.02911 (13)	
O1	0.08196 (6)	0.25832 (11)	0.27072 (9)	0.0306 (3)	
O2	0.16718 (6)	0.44366 (11)	0.17485 (10)	0.0344 (3)	
C1	0.14760 (8)	0.44092 (17)	0.22435 (13)	0.0327 (4)	
C2	0.12987 (8)	0.34853 (16)	0.23120 (13)	0.0294 (4)	
C3	0.10554 (8)	0.34857 (16)	0.27383 (12)	0.0298 (4)	
C4	0.10342 (9)	0.43159 (18)	0.31533 (13)	0.0354 (5)	
H20	0.0882	0.4281	0.3452	0.043*	
C5	0.12496 (9)	0.52060 (18)	0.31050 (14)	0.0388 (5)	
H21	0.1251	0.5769	0.3392	0.047*	
C6	0.14621 (9)	0.52681 (17)	0.26361 (14)	0.0379 (5)	
H22	0.1592	0.5871	0.2587	0.046*	
C7	0.01157 (8)	0.21993 (15)	0.10672 (13)	0.0288 (4)	
C8	0.00137 (8)	0.24372 (17)	0.02814 (13)	0.0321 (4)	
H23	0.0280	0.2449	0.0203	0.038*	
C9	−0.04811 (9)	0.26574 (19)	−0.03860 (15)	0.0391 (5)	
H24	−0.0546	0.2807	−0.0910	0.047*	
C10	−0.08780 (9)	0.26546 (19)	−0.02715 (15)	0.0396 (5)	
H25	−0.1210	0.2798	−0.0719	0.047*	
C11	−0.07809 (9)	0.24373 (19)	0.05141 (15)	0.0397 (5)	
H26	−0.1047	0.2447	0.0594	0.048*	
C12	−0.02885 (9)	0.22066 (18)	0.11754 (15)	0.0363 (5)	
H27	−0.0226	0.2055	0.1698	0.044*	
C13	0.06603 (8)	0.06823 (16)	0.22481 (13)	0.0291 (4)	
C14	0.09125 (8)	0.04158 (18)	0.30946 (14)	0.0347 (5)	
H28	0.1094	0.0897	0.3510	0.042*	
C15	0.08926 (9)	−0.0563 (2)	0.33157 (15)	0.0436 (6)	
H29	0.1058	−0.0733	0.3879	0.052*	
C16	0.06302 (9)	−0.1289 (2)	0.27081 (17)	0.0456 (6)	
H30	0.0637	−0.1952	0.2863	0.055*	
C17	0.03576 (9)	−0.10278 (19)	0.18683 (16)	0.0425 (5)	
H31	0.0168	−0.1508	0.1457	0.051*	
C18	0.03690 (8)	−0.00462 (17)	0.16425 (14)	0.0350 (5)	
H32	0.0179	0.0130	0.1078	0.042*	
C19	0.09695 (8)	0.38005 (16)	0.01862 (13)	0.0302 (4)	
C20	0.07886 (9)	0.32187 (17)	−0.05290 (14)	0.0342 (4)	
H33	0.0962	0.2636	−0.0494	0.041*	
C21	0.03544 (9)	0.34968 (19)	−0.12919 (14)	0.0399 (5)	
H34	0.0239	0.3102	−0.1764	0.048*	
C22	0.00919 (10)	0.4362 (2)	−0.13533 (15)	0.0439 (5)	
H35	−0.0199	0.4550	−0.1866	0.053*	
C23	0.02665 (11)	0.4943 (2)	−0.06468 (16)	0.0471 (6)	
H36	0.0092	0.5524	−0.0685	0.056*	
C24	0.06998 (10)	0.46637 (18)	0.01180 (15)	0.0404 (5)	
H37	0.0812	0.5057	0.0590	0.049*	
C25	0.20242 (8)	0.34292 (16)	0.09881 (13)	0.0321 (4)	
C26	0.19699 (9)	0.39827 (19)	0.03261 (15)	0.0390 (5)	
H38	0.1668	0.4331	−0.0035	0.047*	
C27	0.23669 (10)	0.4015 (2)	0.02033 (16)	0.0468 (6)	
H39	0.2326	0.4378	−0.0244	0.056*	
C28	0.28171 (10)	0.3517 (2)	0.07380 (16)	0.0477 (6)	
H40	0.3078	0.3536	0.0647	0.057*	
C29	0.28847 (10)	0.2984 (2)	0.14149 (17)	0.0451 (6)	
H41	0.3193	0.2662	0.1788	0.054*	
C30	0.24838 (9)	0.29392 (18)	0.15257 (15)	0.0374 (5)	
H19	0.2525	0.2571	0.1971	0.045*	
C31	0.15587 (9)	−0.03061 (17)	0.18758 (15)	0.0372 (5)	
H31A	0.1339	−0.0351	0.2075	0.056*	
H31B	0.1910	−0.0319	0.2344	0.056*	
H31C	0.1494	−0.0863	0.1512	0.056*	
C32	0.08378 (9)	0.05274 (18)	0.02973 (14)	0.0365 (5)	
H32A	0.0886	−0.0096	0.0101	0.055*	
H32B	0.0752	0.1042	−0.0111	0.055*	
H32C	0.0565	0.0458	0.0380	0.055*	
C33	0.18824 (9)	0.07295 (18)	0.09792 (16)	0.0384 (5)	
H33A	0.2227	0.0741	0.1469	0.058*	
H33B	0.1836	0.1271	0.0614	0.058*	
H33C	0.1821	0.0104	0.0690	0.058*	
C34	0.20915 (9)	0.1656 (2)	0.40012 (14)	0.0405 (5)	
H34A	0.1973	0.0975	0.3906	0.061*	
H34B	0.1854	0.2078	0.4028	0.061*	
H34C	0.2422	0.1697	0.4521	0.061*	
C35	0.26675 (9)	0.1240 (2)	0.33529 (15)	0.0441 (5)	
H35A	0.2945	0.1299	0.3932	0.066*	
H35B	0.2784	0.1426	0.3003	0.066*	
H35C	0.2548	0.0560	0.3236	0.066*	
C36	0.25124 (9)	0.32175 (19)	0.36162 (15)	0.0429 (5)	
H36A	0.2819	0.3061	0.4150	0.064*	
H36B	0.2311	0.3688	0.3693	0.064*	
H36C	0.2602	0.3505	0.3256	0.064*	

### Atomic displacement parameters (Å^2^)

**Table:**

	*U*^11^	*U*^22^	*U*^33^	*U*^12^	*U*^13^	*U*^23^
Co1	0.03026 (15)	0.02427 (16)	0.02686 (14)	−0.00035 (10)	0.02072 (12)	−0.00117 (9)
P1	0.0295 (2)	0.0280 (3)	0.0274 (2)	−0.00020 (19)	0.0204 (2)	−0.00030 (19)
P2	0.0387 (3)	0.0245 (3)	0.0315 (3)	−0.0020 (2)	0.0264 (2)	−0.00196 (19)
P3	0.0341 (3)	0.0254 (3)	0.0323 (3)	0.00060 (19)	0.0233 (2)	−0.00147 (19)
P4	0.0304 (3)	0.0329 (3)	0.0284 (3)	−0.0015 (2)	0.0190 (2)	−0.00092 (19)
O1	0.0374 (8)	0.0340 (8)	0.0319 (7)	−0.0016 (6)	0.0263 (6)	−0.0028 (6)
O2	0.0508 (9)	0.0278 (8)	0.0407 (8)	−0.0078 (6)	0.0355 (7)	−0.0064 (6)
C1	0.0422 (11)	0.0335 (12)	0.0331 (10)	−0.0025 (9)	0.0275 (9)	−0.0035 (8)
C2	0.0358 (10)	0.0281 (11)	0.0310 (9)	−0.0004 (8)	0.0227 (8)	−0.0024 (8)
C3	0.0354 (10)	0.0306 (11)	0.0303 (10)	0.0006 (8)	0.0226 (9)	−0.0020 (8)
C4	0.0420 (11)	0.0398 (13)	0.0335 (10)	0.0029 (9)	0.0266 (10)	−0.0038 (9)
C5	0.0518 (13)	0.0338 (12)	0.0393 (11)	0.0004 (10)	0.0306 (11)	−0.0105 (9)
C6	0.0539 (13)	0.0287 (12)	0.0403 (11)	−0.0049 (9)	0.0319 (11)	−0.0076 (9)
C7	0.0337 (10)	0.0267 (11)	0.0317 (10)	0.0012 (8)	0.0217 (9)	0.0006 (7)
C8	0.0360 (10)	0.0338 (11)	0.0335 (10)	0.0044 (8)	0.0238 (9)	0.0021 (8)
C9	0.0433 (12)	0.0431 (14)	0.0345 (11)	0.0082 (10)	0.0239 (10)	0.0046 (9)
C10	0.0341 (11)	0.0425 (14)	0.0395 (12)	0.0080 (9)	0.0189 (10)	0.0024 (9)
C11	0.0364 (11)	0.0449 (14)	0.0475 (13)	0.0049 (9)	0.0293 (10)	0.0025 (10)
C12	0.0383 (11)	0.0420 (13)	0.0383 (11)	0.0025 (9)	0.0273 (10)	0.0038 (9)
C13	0.0306 (9)	0.0317 (11)	0.0346 (10)	−0.0003 (8)	0.0241 (8)	0.0019 (8)
C14	0.0338 (10)	0.0434 (13)	0.0346 (10)	−0.0011 (9)	0.0239 (9)	0.0037 (9)
C15	0.0394 (12)	0.0556 (16)	0.0431 (12)	0.0025 (10)	0.0275 (11)	0.0180 (11)
C16	0.0450 (12)	0.0402 (14)	0.0620 (15)	−0.0015 (10)	0.0362 (12)	0.0134 (11)
C17	0.0436 (12)	0.0371 (13)	0.0551 (14)	−0.0089 (10)	0.0326 (11)	−0.0004 (10)
C18	0.0366 (11)	0.0360 (12)	0.0373 (11)	−0.0039 (9)	0.0235 (9)	0.0009 (9)
C19	0.0429 (11)	0.0233 (10)	0.0358 (10)	0.0012 (8)	0.0291 (9)	0.0022 (8)
C20	0.0460 (12)	0.0284 (11)	0.0360 (11)	0.0049 (9)	0.0277 (10)	0.0017 (8)
C21	0.0505 (13)	0.0389 (13)	0.0351 (11)	0.0029 (10)	0.0268 (10)	0.0032 (9)
C22	0.0474 (13)	0.0466 (15)	0.0408 (12)	0.0109 (10)	0.0266 (11)	0.0135 (10)
C23	0.0612 (15)	0.0386 (14)	0.0505 (14)	0.0178 (11)	0.0367 (13)	0.0105 (11)
C24	0.0567 (14)	0.0320 (12)	0.0437 (12)	0.0057 (10)	0.0348 (11)	0.0023 (9)
C25	0.0434 (11)	0.0290 (11)	0.0368 (11)	−0.0070 (8)	0.0303 (10)	−0.0065 (8)
C26	0.0483 (12)	0.0414 (13)	0.0403 (11)	−0.0050 (10)	0.0327 (11)	−0.0009 (9)
C27	0.0586 (15)	0.0553 (16)	0.0457 (13)	−0.0136 (12)	0.0410 (12)	−0.0046 (11)
C28	0.0502 (14)	0.0625 (17)	0.0498 (14)	−0.0168 (12)	0.0399 (12)	−0.0125 (12)
C29	0.0441 (13)	0.0511 (15)	0.0517 (14)	−0.0058 (11)	0.0338 (12)	−0.0065 (11)
C30	0.0424 (12)	0.0391 (13)	0.0415 (12)	−0.0049 (9)	0.0300 (10)	−0.0015 (9)
C31	0.0412 (11)	0.0297 (12)	0.0461 (12)	0.0029 (9)	0.0276 (10)	0.0026 (9)
C32	0.0434 (12)	0.0345 (12)	0.0364 (11)	−0.0015 (9)	0.0252 (10)	−0.0054 (9)
C33	0.0479 (12)	0.0327 (12)	0.0510 (13)	0.0006 (9)	0.0377 (11)	−0.0035 (10)
C34	0.0399 (11)	0.0514 (15)	0.0330 (11)	−0.0042 (10)	0.0220 (10)	0.0025 (10)
C35	0.0371 (11)	0.0549 (16)	0.0392 (12)	0.0095 (10)	0.0206 (10)	0.0031 (11)
C36	0.0427 (12)	0.0445 (14)	0.0373 (12)	−0.0095 (10)	0.0195 (10)	−0.0021 (10)

### Geometric parameters (Å, °)

**Table:**

Co1—C2	1.961 (2)	C16—C17	1.384 (4)
Co1—P1	2.1278 (7)	C16—H30	0.9300
Co1—P2	2.1337 (6)	C17—C18	1.386 (3)
Co1—P3	2.1819 (7)	C17—H31	0.9300
Co1—P4	2.2180 (13)	C18—H32	0.9300
P1—O1	1.6801 (15)	C19—C20	1.393 (3)
P1—C13	1.824 (2)	C19—C24	1.392 (3)
P1—C7	1.839 (2)	C20—C21	1.385 (3)
P2—O2	1.6829 (15)	C20—H33	0.9300
P2—C19	1.834 (2)	C21—C22	1.385 (3)
P2—C25	1.839 (2)	C21—H34	0.9300
P3—C31	1.834 (2)	C22—C23	1.382 (4)
P3—C32	1.835 (2)	C22—H35	0.9300
P3—C33	1.837 (2)	C23—C24	1.386 (4)
P4—C36	1.829 (2)	C23—H36	0.9300
P4—C34	1.835 (2)	C24—H37	0.9300
P4—C35	1.836 (2)	C25—C30	1.385 (3)
O1—C3	1.398 (3)	C25—C26	1.395 (3)
O2—C1	1.390 (2)	C26—C27	1.394 (3)
C1—C6	1.386 (3)	C26—H38	0.9300
C1—C2	1.389 (3)	C27—C28	1.371 (4)
C2—C3	1.397 (3)	C27—H39	0.9300
C3—C4	1.387 (3)	C28—C29	1.389 (4)
C4—C5	1.396 (3)	C28—H40	0.9300
C4—H20	0.9300	C29—C30	1.391 (3)
C5—C6	1.390 (3)	C29—H41	0.9300
C5—H21	0.9300	C30—H19	0.9300
C6—H22	0.9300	C31—H31A	0.9600
C7—C8	1.392 (3)	C31—H31B	0.9600
C7—C12	1.397 (3)	C31—H31C	0.9600
C8—C9	1.388 (3)	C32—H32A	0.9600
C8—H23	0.9300	C32—H32B	0.9600
C9—C10	1.382 (3)	C32—H32C	0.9600
C9—H24	0.9300	C33—H33A	0.9600
C10—C11	1.392 (3)	C33—H33B	0.9600
C10—H25	0.9300	C33—H33C	0.9600
C11—C12	1.383 (3)	C34—H34A	0.9600
C11—H26	0.9300	C34—H34B	0.9600
C12—H27	0.9300	C34—H34C	0.9600
C13—C18	1.396 (3)	C35—H35A	0.9600
C13—C14	1.400 (3)	C35—H35B	0.9600
C14—C15	1.386 (3)	C35—H35C	0.9600
C14—H28	0.9300	C36—H36A	0.9600
C15—C16	1.381 (4)	C36—H36B	0.9600
C15—H29	0.9300	C36—H36C	0.9600
			
C2—Co1—P1	76.63 (6)	C15—C16—C17	119.8 (2)
C2—Co1—P2	78.62 (6)	C15—C16—H30	120.1
P1—Co1—P2	131.45 (3)	C17—C16—H30	120.1
C2—Co1—P3	171.95 (6)	C16—C17—C18	119.7 (2)
P1—Co1—P3	97.37 (3)	C16—C17—H31	120.2
P2—Co1—P3	102.04 (3)	C18—C17—H31	120.2
C2—Co1—P4	87.75 (6)	C17—C18—C13	121.1 (2)
P1—Co1—P4	111.03 (3)	C17—C18—H32	119.4
P2—Co1—P4	108.97 (3)	C13—C18—H32	119.4
P3—Co1—P4	99.52 (3)	C20—C19—C24	118.4 (2)
O1—P1—C13	97.69 (8)	C20—C19—P2	119.48 (16)
O1—P1—C7	100.23 (9)	C24—C19—P2	121.96 (17)
C13—P1—C7	99.71 (9)	C21—C20—C19	120.9 (2)
O1—P1—Co1	107.20 (6)	C21—C20—H33	119.6
C13—P1—Co1	125.25 (7)	C19—C20—H33	119.6
C7—P1—Co1	121.73 (7)	C22—C21—C20	120.2 (2)
O2—P2—C19	99.43 (9)	C22—C21—H34	119.9
O2—P2—C25	96.95 (9)	C20—C21—H34	119.9
C19—P2—C25	100.04 (10)	C23—C22—C21	119.4 (2)
O2—P2—Co1	106.81 (6)	C23—C22—H35	120.3
C19—P2—Co1	119.78 (7)	C21—C22—H35	120.3
C25—P2—Co1	128.16 (8)	C22—C23—C24	120.5 (2)
C31—P3—C32	99.98 (11)	C22—C23—H36	119.8
C31—P3—C33	99.52 (11)	C24—C23—H36	119.8
C32—P3—C33	98.87 (11)	C23—C24—C19	120.6 (2)
C31—P3—Co1	117.78 (8)	C23—C24—H37	119.7
C32—P3—Co1	116.75 (8)	C19—C24—H37	119.7
C33—P3—Co1	120.15 (8)	C30—C25—C26	118.4 (2)
C36—P4—C34	98.70 (11)	C30—C25—P2	118.83 (16)
C36—P4—C35	98.68 (13)	C26—C25—P2	122.72 (18)
C34—P4—C35	97.73 (12)	C27—C26—C25	120.2 (2)
C36—P4—Co1	115.83 (9)	C27—C26—H38	119.9
C34—P4—Co1	117.01 (8)	C25—C26—H38	119.9
C35—P4—Co1	124.27 (8)	C28—C27—C26	120.4 (2)
C3—O1—P1	107.12 (12)	C28—C27—H39	119.8
C1—O2—P2	109.21 (13)	C26—C27—H39	119.8
C6—C1—O2	120.65 (19)	C27—C28—C29	120.3 (2)
C6—C1—C2	123.14 (19)	C27—C28—H40	119.9
O2—C1—C2	116.21 (18)	C29—C28—H40	119.9
C1—C2—C3	115.76 (18)	C28—C29—C30	119.0 (3)
C1—C2—Co1	121.87 (15)	C28—C29—H41	120.5
C3—C2—Co1	122.29 (16)	C30—C29—H41	120.5
C4—C3—C2	123.6 (2)	C25—C30—C29	121.6 (2)
C4—C3—O1	121.24 (18)	C25—C30—H19	119.2
C2—C3—O1	115.15 (17)	C29—C30—H19	119.2
C3—C4—C5	117.47 (19)	P3—C31—H31A	109.5
C3—C4—H20	121.3	P3—C31—H31B	109.5
C5—C4—H20	121.3	H31A—C31—H31B	109.5
C6—C5—C4	121.3 (2)	P3—C31—H31C	109.5
C6—C5—H21	119.3	H31A—C31—H31C	109.5
C4—C5—H21	119.3	H31B—C31—H31C	109.5
C1—C6—C5	118.3 (2)	P3—C32—H32A	109.5
C1—C6—H22	120.8	P3—C32—H32B	109.5
C5—C6—H22	120.8	H32A—C32—H32B	109.5
C8—C7—C12	118.6 (2)	P3—C32—H32C	109.5
C8—C7—P1	119.80 (16)	H32A—C32—H32C	109.5
C12—C7—P1	121.56 (16)	H32B—C32—H32C	109.5
C9—C8—C7	120.7 (2)	P3—C33—H33A	109.5
C9—C8—H23	119.6	P3—C33—H33B	109.5
C7—C8—H23	119.6	H33A—C33—H33B	109.5
C10—C9—C8	120.0 (2)	P3—C33—H33C	109.5
C10—C9—H24	120.0	H33A—C33—H33C	109.5
C8—C9—H24	120.0	H33B—C33—H33C	109.5
C9—C10—C11	120.0 (2)	P4—C34—H34A	109.5
C9—C10—H25	120.0	P4—C34—H34B	109.5
C11—C10—H25	120.0	H34A—C34—H34B	109.5
C12—C11—C10	119.9 (2)	P4—C34—H34C	109.5
C12—C11—H26	120.1	H34A—C34—H34C	109.5
C10—C11—H26	120.1	H34B—C34—H34C	109.5
C11—C12—C7	120.7 (2)	P4—C35—H35A	109.5
C11—C12—H27	119.6	P4—C35—H35B	109.5
C7—C12—H27	119.6	H35A—C35—H35B	109.5
C18—C13—C14	118.3 (2)	P4—C35—H35C	109.5
C18—C13—P1	120.03 (16)	H35A—C35—H35C	109.5
C14—C13—P1	121.19 (17)	H35B—C35—H35C	109.5
C15—C14—C13	120.1 (2)	P4—C36—H36A	109.5
C15—C14—H28	119.9	P4—C36—H36B	109.5
C13—C14—H28	119.9	H36A—C36—H36B	109.5
C16—C15—C14	120.7 (2)	P4—C36—H36C	109.5
C16—C15—H29	119.6	H36A—C36—H36C	109.5
C14—C15—H29	119.6	H36B—C36—H36C	109.5

## References

[bb1] Aydin, J., Kumar, K. S., Erilsson, L. & Szabo, K. J. (2007). *Adv. Synth. Catal.* **349**, 2585–2594.

[bb2] Bedford, R. B., Betham, M., Blake, M. E., Coles, S. J., Draper, S. M., Husthouse, M. B. & Scully, P. N. (2006). *Inorg. Chim. Acta*, **359**, 1870–1878.

[bb3] Boom, M. & Milstein, D. (2003). *Chem. Rev.* **103**, 1759–1792. 10.1021/cr960118r 12744693

[bb4] Bruker (1997). *SMART* and *SAINT* Bruker AXS Inc., Madison, Wisconsin, USA.

[bb5] Burnett, M. N. & Johnson, C. K. (1996). *ORTEPIII* Report ORNL-6895. Oak Ridge National Laboratory, Tennessee, USA.

[bb6] Farrugia, L. J. (1997). *J. Appl. Cryst.* **30**, 565.

[bb7] Gomez-Benitez, V., Baldovino-Pantaleon, O., Herrera-Alvarez, C., Toscano, R. A. & Morales-Morales, D. (2006). *Tetrahedron Lett.* **47**, 5059–5062.

[bb8] Kimura, T. & Uozumi, Y. (2006). *Organometallics*, **25**, 4883–4887.

[bb9] Sheldrick, G. M. (2004). *SADABS* University of Göttingen, Germany.

[bb10] Sheldrick, G. M. (2008). *Acta Cryst.* A**64**, 112–122. 10.1107/S0108767307043930 18156677

[bb11] Xu, G., Sun, H. & Li, X. (2009). *Organometallics*, **28**, 6090–6095.

